# Identifying and Shifting Disempowering Paradigms for Families of Children With Disability Through a System Informed Positive Psychology Approach

**DOI:** 10.3389/fpsyg.2021.663640

**Published:** 2021-12-23

**Authors:** Sylvana Mahmic, Margaret L. Kern, Annick Janson

**Affiliations:** ^1^Plumtree Children’s Services, Sydney, NSW, Australia; ^2^School of Education, Western Sydney University, Sydney, NSW, Australia; ^3^Centre for Wellbeing Science, Melbourne Graduate School of Education, The University of Melbourne, Melbourne, VIC, Australia; ^4^Centre for Cross Cultural Research, Victoria University of Wellington, Wellington, New Zealand

**Keywords:** wellbeing literacy, systems-informed positive psychology, disability, early intervention, family-centered, capacity building, parent empowerment, paradigm shifts

## Abstract

Despite the emergence of socio-ecological, strength-based, and capacity-building approaches, care for children with disability remains primarily grounded in a deficit-based perspective. Diagnoses and interventions primarily focus on what children and families cannot do, rather than what might be possible, often undermining the competence, mental health, and functioning of both the children and their families. We first critically examine typical approaches to disability care for families of young children, describe the importance of a systems-informed positive psychology (SIPP) approach to care, and identify the existence of two dominant paradigms, *disability is a disadvantage* and *experts know best*. Then, we present a case study investigating families’ experiences with these two paradigms and whether shifts to alternative perspectives could occur through participation in a SIPP-based program co-designed by professionals and families. Of program participants, nine parents and five early intervention professionals participated in two separate focus groups, and ten e-books were randomly selected for review. Thematic analysis of the e-books and focus group data identified two primary themes representing alternative perspectives that arose through the intervention: *we will start with our strengths* and *we’ve got this*. Participant comments indicated that they developed a greater sense of hope, empowerment, engagement, and wellbeing, enabled by embedding wellbeing concepts and practices in their routines and communications with their children. We suggest that benefits arose in part from the structure of the program and the development of wellbeing literacy in participants. While care needs to be taken in generalizing the results, the case study provides clear examples of shifts in perspectives that occurred and suggests that the incorporation of SIPP principles within early intervention approaches provides a potential pathway for shifting the problematic paradigms that dominate disability care.

## Introduction

Over one billion people worldwide have one or more disabilities (World Health Organization, 2015), with health, developmental, social, economic, and functional consequences not only on those with the disability, but also on families, schools, workplaces, and others in the community. Despite the emergence of socioecological models that emphasize the fit between the person and the environment (e.g., Shakespeare, 2013, 2016), research and practice – including language, diagnoses, funding, and interventions – still primarily focus on what children and families cannot do, rather than what might be possible. These traditional approaches do not serve those with disabilities or their families well, often with the unintended consequence of undermining the competence, mental health, and functioning of both the children and their families.

Parallels can be seen in the field of psychology. Across the 20th century, research and care primarily focused on deficit and dysfunction, developing a range of approaches and treatments to treat mental disorders. Yet over the past few decades, work within positive psychology has suggested the potential and need to add specific focus on understanding and cultivating human strengths, potential, and thriving. Applied to disability, the positive psychology perspective suggests that even as disability brings a number of challenges, it also brings a number of possibilities and opportunities (Blacher et al., 2013a; Wehmeyer, 2013). Further, although early work in positive psychology primarily focused on individual wellbeing and flourishing, more recent theory and practice increasingly incorporate systems-informed principles [i.e., systems-informed positive psychology (SIPP); Kern et al., 2020; see also Lomas et al., 2020], akin to the family-systems approaches that have arisen within disability care. A SIPP perspective appears within early intervention efforts that focus on family strengths, such as the peer led *Now and Next* parenting program (Moore et al., 2018), but remains on the fringes, rather than being central to care.

Could there be potential for an alternative approach to mainstream disability care that is more family-centered and strengths focused? From a SIPP perspective, leveraging such change requires not only changing policies and structures around care, but also revealing and shifting the deeper paradigms and collective mindsets from which those policies and structures arise (Meadows, 1999; Kern et al., 2020). In this article, we first describe typical approaches to disability care for children and their families. Second, we describe the SIPP perspective, pointing to principles that are relevant to and provide a theoretical foundation for the focus of and approaches used in the current study. Third, we identify two paradigms apparent in contemporary disability care and consider the impact that these paradigms have. Fourth, we present a case study investigating families’ perspectives could shift through participation in a capacity-building program informed by SIPP principles and co-designed by professionals and families. Although the case study is not representative of broader samples, it begins to give voice to the experiences and perspectives of families who are deeply impacted by but often disempowered by the dominant approaches and paradigms driving disability research and practice. Fifth, we consider potential mechanisms for shifting existing perspectives. Finally, we consider implications for research, practice, and broader systemic aspects that impact upon family care and functioning.

### Approaches to Disability Care

Current models of disability care for families of young children are influenced by two contrasting perspectives rooted in the 1960s: a traditional biomedical approach and a holistic biopsychosocial approach. In the traditional biomedical approach, assessment procedures diagnose dysfunction and identify cognitive, emotional, behavioral, sensory, physical, and developmental issues to guide treatment (Rosenbaum and Gorter, 2012). Rooted in a deficit-based model, the professional serves as an expert who identifies and solves problems for families, using their training and expertise (McWilliam and Scott, 2001; Dunst and Trivette, 2009; Dunst, 2012). Professionals directly intervene with children to address issues arising from the disability, or parents are taught to use professionally identified therapeutic practices to support their child’s development (Sukkar et al., 2016). Although the traditional biomedical approach can be effective for diagnosing issues, treating acute symptoms, and guiding funding decisions when limited resources are available, when families continually defer to professional expertise, their parenting role is diminished, compromising their self-efficacy (Dunst et al., 2019).

In contrast, the biopsychosocial approach holistically identifies the strengths and challenges for the child, family, and environment, addressing the family as a dynamic system. Care includes specific focus on the role of parents/carers to support their child’s development, along with aspects of the home environment that might be altered to better support the child and family as a whole (Dunst, 2016; Dunst and Espe-Sherwindt, 2016). A family-systems approach regards the role of families as critical in supporting the development of children, as families have the primary responsibility to embed learning opportunities in daily family life. Families are also part of their own kinship networks, along with broader community and societal systems, including health and education organizations, and together these have significant impact on the lives of children with disability and their families. Early intervention support for children and families, therefore, occurs across and is influenced by familial and broader systems.

Family-systems models have evolved to include a greater emphasis on family capacity building. For instance, Dunst and Trivette (2009) integrated various early intervention approaches that build family capabilities before additional symptoms and problems occur, which they contrasted with traditional deficit-based treatment approaches. A meta-analysis by Dunst et al. (2019) demonstrated that capacity-building practices increase parent self-efficacy, which in turn improve parent and child interactions, with corresponding positive child outcomes. Still, although early intervention capacity-building approaches have been used internationally across a variety of health and human services, the traditional deficit-focused approach to disability care remains prevalent.

### Systems-Informed Positive Psychology

Systems-informed positive psychology “explicitly incorporates principles and concepts from the systems sciences into positive psychology theory, methodologies, practices, and discourse to optimize human social systems and the individuals within them” (Kern et al., 2020, p. 505). SIPP assumes that people are inter-dependent with the people and environments in which they reside, with each element dynamically interacting with and influencing the others. As such, care for a child with disability requires attention to the family, home environment, professional care, and the interactions among these elements. SIPP aims to cultivate optimal functioning and development for all people, regardless of background or ability. SIPP calls for equitable consideration of different perspectives, expertise, and values. For instance, although professionals bring expertise in terms of clinical training and tools and strategies for care, parents/carers bring expertise about their child’s strengths, challenges, and behavioural patterns.

Systems-informed positive psychology also points to the importance of simplexity – embracing the dialectical tension between the complexity of disability care and the need for simple places to effectively intervene (Kern et al., 2020). Levers of change dynamically change at different points across the disability care, including policies set around care, structures of informational flow, goals at different levels within the system (including goals for government, society, professionals, and families), policies impacting upon care, and the deeper mindsets and paradigms that drive the disability care system as a whole (Meadows, 1999). We suggest that the dominance of the traditional approach arises from the unacknowledged yet powerful paradigms that are embedded across disability care.

A societal paradigm is an idea or a set of shared unstated or unverified assumptions upon which complex social structures are built (Meadows, 1999). It is very difficult to change these foundational paradigms, largely because they are so deeply embedded within social structures. Still, paradigms can change, in part by repeatedly and consistently pointing out anomalies and failures in current paradigms to people who have enough of an open mind to test unspoken assumptions, combined with an ability to influence the system that they are a part of (Meadows, 1999). From a systems-informed perspective, such change necessarily begins with shining a light on the existing paradigms, making the invisible visible.

### Paradigms in Disability Care

We suggest that despite efforts for reform, the majority of disability care remains grounded in traditional, deficit-based, and expert-focused approaches to care, due in part to often unacknowledged paradigms, which impact upon how professionals are trained, disability-related policies, funding structures, approaches to care, and stakeholder expectations. We focus here on two specific paradigms that occur within the child disability sector: *disability is a disadvantage* and *experts know best*.

#### Disability Is a Disadvantage

A first paradigm driving much of disability care is the assumption that *disability is a disadvantage*. For more than half a century, research has focused on the negative impacts of children with a disability on their family (Blacher et al., 2013a). From this perspective, when a child is diagnosed with a disability, parents are assumed to experience grief about losing the healthy child they had expected and subsequently experience chronic sorrow through the ongoing challenges arising from raising a disabled child. Disability is viewed as a tragedy, and the disabled child as flawed. Despite this perspective being largely disempowering and potentially harmful for families and the child, the grief and chronic sorrow perspective has remained largely unchallenged (Allred, 2015).

The *disability as a disadvantage* paradigm further appears in the advent of antenatal testing and the practice of selective termination, which attempt to prevent disabilities from developing or even preventing a potentially disabled child from entering the world, implicitly making value-ladened judgments about human worth (Shakespeare and Hull, 2018). The Australian immigration policy discriminates against migrants who have a child with a disability on the basis of economic cost to the government (Yu, 2014). Disability is seen as an unnecessary social cost rather than as a valued part of human experience. These issues are ethically and politically contentious, and remain unresolved (Shakespeare, 1998).

We acknowledge that this paradigm does not characterize all of disability research and care. For example, various studies over the past two decades have examined the positive impacts of a child with a disability (e.g., Blacher and Baker, 2007; Blacher et al., 2013b), influenced largely by the disability rights movement. Such studies advocate for a socioecological model of disability, which asserts that society was structured for people who do not have disability; thus, the existence of many of the challenges of disability arise from society not being properly structured for the needs of those with disability, rather than an inherent problem with the individuals themselves (Shakespeare, 2016). Still, while progress has been made by disability activists and the broader community, significant systemic barriers remain (National People with Disabilities and Carer Council, 2010).

It is in this context that families of young children with disability must navigate the immediate needs facing their child and family. Parenting a child with a disability presents unexpected and sometimes challenging experiences. These can lead to stress, mental health disorders, health issues, strain on family relationships, marital breakdown, financial pressures, and unemployment (Reichman et al., 2008; Bourke-Taylor et al., 2010, 2011; Bhopti, 2017). These pressures are exacerbated by deficit-focused systems, which also view disability as a disadvantage, and which influence the way families are supported by professionals.

#### Experts Know Best

A second paradigm that drives much of disability care is that *experts know best*. Despite the existince of family-centered approaches that aim to empower families, professionals still dominate the early intervention process (Dunst, 2016). Families are unsurprisingly at risk of deferring to professional expertise and experience in the early diagnosis period (Lee, 2015). The initial process of diagnosis, how that information is explained, and the support – or lack thereof – is in itself a significant experience for many families (Murray, 2000). Families face challenges with making sense of and understanding how to support their child, navigate various systems, and manage difficult processes for attaining funding and resources. They need information about how to select interventions for their child, services providers, and participating in effective planning processes (Tracey et al., 2018). They may not understand nor value that their family has the most significant impact on their child (Mahoney and Perales, 2011). Various factors – including the parents’ education level, socioeconomic status, race, ethnicity, language, number of children, and immigration status – place vulnerable families at even greater risk for deferring to experts. The challenges of the diagnosis period, combined with the number of decisions that must be made can disempower families, often result in families placing control over care in the hands of the professional, with positive or negative experiences and outcomes dependent upon the professionals that the family has or is granted access to.

The deference to professionals can be further accentuated by the professionals themselves. Families are often not viewed as equally contributing partners in their child’s early intervention, despite collaborative partnerships being a critical feature of family-centered practice (Summers et al., 2007; Espe-Sherwindt, 2008). Professionals can find it challenging to see the family as bringing a complementary expertise, arising from their own knowledge of and experiences with the child, deferring to their own academically grounded knowledge (Dodd et al., 2009), with training implicitly reinforcing the superior expertise of the professional (Zhang and Bennett, 2003). As such, within the planning process, professionals often dominate the conversation, causing parents to feel left out of important decisions. Bureaucratic requirements overtake opportunities for families to express their choices and priorities, and in doing so, the voice of professionals, as expert and authority, is prioritized over that of the family (Lee, 2015).

The paradigm of experts knowing best is due in part to the operational indicators of effective family-professional partnerships not being fully understood (Dunst and Dempsey, 2007). There remain gaps between research around family-centered approaches and practice (Dunst, 2007). For professionals who do want to support more equal partnerships, they face challenges with the high costs of delivering quality family-centered services, limited understanding and support from colleagues and managers (Espe-Sherwindt, 2008), and funding systems that incentivizes expert-focused models (McDonald et al., 2016; Moore et al., 2019). Evidence-informed decision-making frameworks would assist families in this period but are not always available (Moore, 2016). While families need the expertise of professionals, the provision of services must be family-centered to remain focused on empowerment of the family.

## Leveraging Positive Change: a Case Study

We next present a case study investigating whether participation in a capacity-building program built upon SIPP principles and co-designed by researchers, families, and professionals might alter participants’ perspectives around disability care. The case study draws on data collected as part of an action research project investigating the impact of individual funding on families of young children with disability [*cf.* Mahmic (2021) for description and results of the full project]. The project took place in Australia, where individual funding is allocated to individuals by the government so that they can purchase needed services.

The reconnaissance phase of the action research identified active parental/carer participation in planning and decision-making and the use of capacity-building approaches as key priorities, and consequently became the focus in the remaining two cycles. The current study draws on data collected during the second cycle, which involved the co-design of an intervention by professionals and families. All procedures were approved by the Western Sydney University Human Research Ethics Committee (#H 9717).

### The Intervention

The intervention used in the second cycle of the action research project included two components: a novel planning tool and an electronically based portfolio (e-book). Both the planning tool and e-books aimed to build family participation and capacity throughout the intervention, providing greater voice and involvement in the care process.

First, a prototype for a novel planning tool was co-designed with the professionals. The professionals subsequently trialed the tool with the parents/carers. The tool was refined through multiple iterations over several months, until professionals and parents/carers indicated that the tool was useful.^1^ The final tool took approximately an hour for the parents/carers to complete, with minimal guidance from the professional. Parents/carers identified and prioritized goals using a reflective process, where they were prompted to make a selection from 50 images and then were guided toward generating goals for themselves, their family, and their child.

Second, an electronically based portfolio (e-book) was co-designed with the professionals, which provided a structured approach to allow participants to record their goals, activities, successes, and challenges using a combination of modalities (e.g., written text, photos and videos; see Supplementary Material 1 for example entries). This multimedia approach eliminated potential language barriers for culturally diverse parents as they could independently record progress in their e-books using their preferred format thereby, allowing them to participate according to their confidence and capability with English.

The e-books focused on five themes, with each theme acting as a chapter: choosing goals and making plans, gathering information, organizing supports, learning from experiences, and next step thinking. In chapter 1, parents/carers recorded goals for their child, their family, and themselves. In chapter 2, they recorded how to gather and/or what information was needed to achieve their goals. In chapter 3, they recorded the various supports they needed to achieve their goals. In chapter 4, they reflected on what they had learnt throughout the intervention. In chapter 5, they planned their next steps to keep planning and achieving outcomes for their child and family.

The professionals coached the parents to regularly record their progress in the e-book, with opportunities for additional entries and contributions between sessions. The first session involved identifying and recording goals in the e-book (chapter 1). Sessions were then individualized and guided by reflections that parents/carers recorded in the e-book, both during sessions with the professionals and during interim periods.

### Case Study Participants

Fifty-one parents/carers and five professionals participated in the intervention using the planning tool and the e-books, providing consent for their e-books to be used for research purposes. As participants came from diverse backgrounds, many of the text-based entries were in English, but some e-books included a range of language. Ten e-books were randomly selected for analysis, all of which only contained English entries and thus may not represent parents/carers less comfortable with English. The e-books ranged between 15 and 67 pages in length.^2^

All families and professionals were also invited to participate in a focus group discussing their experiences with the intervention, with nine parents/carers and all five professionals agreeing to participate and consenting to de-identified information being used for research purposes. To protect potential identification of individual participants, demographic information for program participants (and subsequently the selected e-books) was not collected, and only limited demographic information was collected for the 14 focus group participants. As such, while the data presented here explore the experiences of a particular set of people, the data are not meant to represent families of children with disability and professionals more broadly.

The two separate focus groups for parents/carers and professionals lasted 77 and 94 min, respectively. The parent/carer group included eight mothers and one grandmother. One mother was from an Anglo-Saxon background; the remainder was from culturally and linguistically diverse backgrounds and spoke English as their second language. We did not directly ask about socioeconomic status, but the socioeconomic level in the region is average or above average compared to the national distribution (Australian Bureau of Statistics, 2016). Participants identified their cultural heritage as Vietnamese, Italian, Egyptian, Korean, and Chinese. All the families had between 1 and 4 years of experience with using various individual funding schemes to support them to reach identified goals. Their children had been diagnosed with Autism, Down Syndrome, or developmental delay and were between three and 7 years of age.

The professional group included five women, who had between five and 15 years of experience in the fields of social work, psychology, speech therapy, counseling, and bilingual family work. Four of the professionals were recruited specifically to co-design and deliver this new program and the bilingual worker was a long-term employee of the organization.

Each group was asked 11 semi-structured questions regarding their experience of the intervention by two trained interviewers (see Supplementary Material 2 for the interview questions). Interviews were recorded and transcribed, resulting in 72 pages of transcribed text.^3^

### Analytic Procedure

Thematic analysis of the focus groups and e-books used an inductive approach to generate codes and categories (Saldaña, 2016). The process was completed over a four-month period and included familiarization with the data through repeated readings, coding, and generation of themes (Clarke and Braun, 2006). The process involved two cycles of coding. The first author manually completed the first cycle, which involved printing the transcribed data, cutting the printed pages into single sentences/phrases, and manually sorting into themes. Analytic memos were recorded, which provided reflections on the responses from both the professional and family perspectives, emergent themes, and future directions for the research.

This was followed by a second cycle, completed by the first and third authors, which used focused coding. Responses were placed into an electronic document and organized into a table, and specific quotes were collated into themes and sub-themes based on the three-column method (Liamputtong, 2013). Representative quotes from participants in support of each sub-theme were identified and reported below.

We coded e-books and interview data separately, and then identified convergent and inconsistent themes across the two sources, with final themes representing areas of convergence across the two sources (Miles et al., 2020). Data from the e-books were weighted more heavily than the focus group data and field notes, as they represented richer and thicker descriptions generated by the families over a longer period (i.e., 3 to 6 months) than the focus group data and were randomly selected from the broader set of 51 parents/carers.

### Results

Thematic analyses of the e-books and focus group data resulted in two primary themes that parents/carers experienced through participation in the intervention: *we will start with our strengths* and *we’ve got this*. These broader themes included a number of sub-themes, which we describe below with examples from the focus groups and e-book data. Parent/carer perceptions of experiences with the existing disability paradigms compared to the perspectives developed through the intervention are considered, along with perceived impacts upon their own, their family, and their child’s wellbeing.

#### Theme 1: We Will Start With Our Strengths

The first theme that arose was *we will start with our strengths*. This appeared across three sub-themes: (a) my child’s strengths, (b) setting a vision and making a plan, and (c) working to achieve goals and celebrate success.

##### My Child’s Strengths

A first sub-theme focused on identifying the child’s strengths. The intervention commenced with an activity in which families documented their child’s strengths and interests. Participants were asked to select from 20 small toys during a quiet reflection process and then were invited to connect their child’s strengths and interests with one of the supplied toys/characters. They were asked the reason for this selection, which provided an opportunity to talk spontaneously about their child’s personality. Parents/carers then recorded these strengths and interests into the first chapter of their e-book, along with photos of their child.

All participants were able to identify their child’s strengths. In the focus groups, parents/carers reflected upon the focus on strengths as counterintuitive, as their instinct was to talk with professionals about the developmental areas in which their child was experiencing difficulty. For example, one parent noted: “*because our children have special needs, our brains seem to have become focused and programmed in fixing (things).*” Another parent noted “*We focused on the things that kids can’t do too, like they can’t talk so we focus on that and we don’t stop to think, how about doing dancing or art classes or music classes?*” In contrast, the intervention provided a structure for families to start the intervention with their child’s strengths and then were reminded about these strengths each time they opened their e-books. For example, one mother selected a superhero character and portrayed her child visually as a superhero. She wrote in her e-book: “*He looks strong and ready for any challenges he might face!*”

Professionals similarly noticed the shift that arose by focusing on strengths. For instance, one professional noted:

*“I’ve found that as a therapist participating in the facilitation that handing the family a toy or the objects and then, them engaging in selecting the toy, focused the family on the child, their child’s strengths, and playful attributes. So it got to that positive strengths based, you know selecting the kind of agenda from the start. That was one of the things that struck me as a therapist, cause you know, you wouldn’t have arrived at that so quickly or it wouldn’t have elicited that so readily in the traditional approach.”*

##### Setting a Vision and Making a Plan

A second sub-theme pointed to families independently developing a plan in which they set their own vision. Of the 10 e-books reviewed, every participant was able to document a vision for their family, along with specific goals aligned with that vision, expressed in their own words. This included goals that they could achieve by themselves without the involvement of professionals. The e-books further showed that the process highlighted their own strengths and capabilities and increased motivation and agency to work on the goals.

In the focus group, parents/carers indicated that they enjoyed the process and found it a refreshing approach, as it was perceived to be fun and fast tracked their thought process involved with planning. For example, one parent described the process in the following manner:

*“So if someone says to you “Oh tell me your goals” and you’ll be saying “Oh my God, where do I start?” and you’ve got all sorts of things going around in your head. But with the cards, like you look at them and go “Oh yeah we want …” and everyone looks at the cards and gets something different out of them. But they were good; they sort of started me thinking. And I think we came up with ideas out of the cards that we might not have even thought of ourselves or we might have thought about later after we left.”*

Similarly, one professional reflected:

*“I think it’s a combination not only of the pictorial cards but of them having to write the goal, so that it’s both senses, like visual and then writing. I think that is where the power comes because then they have to verbalise it one way or another, whether it’s saying what it is or writing it down … they’ve got to really conceptualize it much clearer.”*

The process prompted parents/carers to identify family and personal goals, in addition to goals for their child. These included goals that described their desire for more time to relax, look after their own health, or spend time with partners and friends and to engage in hobbies, education, or employment. For instance, one parent noted:

*“I find that instead of just focusing on the child, you focus on the family as a whole. And you have that kind of holistic approach to life, the whole family, instead of just focusing on the child because I could see that my second child will suffer if we focus too much on the first one … with that, the physical thing that you put together, I think that you sort of organize the thought in your mind as well, of where you’re heading to and how you’ll get there.”*

A professional similarly saw the value of the goal process, noting

*“I don’t think we were able to progress (this mothers) child’s goals sufficiently because we got caught up in what she had done for herself. So she progressed her personal goals, that she’d found amazing creativity for herself that she had forgotten about and not used.”*

##### Working to Achieve Goals and Celebrating Success

A third sub-theme reflected working toward achieving goals and the celebration of success. The e-books not only gave participants a sense of progression, but also opportunities to celebrate their successes. Participants reported enjoying using the e-books, as it helped them to document their progress on goals in written and visual format, record ideas, and celebrate successes, which helped to keep them focused on progress and outcomes leaving them more motivated. For instance, one parent noted:

*“I found the book, you know when you talk about the goals, I found the book really good to separate all the things out … I think I’ve had so much going on in my head that it was all just a little bit hectic and crazy … I get stressed out about everything. So with me having the book, because I think because I’m visual, I’ve got it all set out.”*

Participants also stayed connected to the goals that they had identified throughout the sessions and were then able to direct their resources and attention toward achieving those goals. Parents/carers identified that it was beneficial to have time and a process to help them to figure all this out for themselves. For instance, one parent noted:

*“Brainstorming ways of making the goals happen is helpful. The steps to achieving the goals we have put down make it easy for me to understand the process and to meet the goals. Having created the e-book has helped me to understand having plans and goals is achievable.”*

Professionals similarly saw the value of working toward goals and the role of success. For instance, one professional noted:

*“Many families have goals, have goals for their child that in the end they never really put into practice but when this program came it actually gave them a chance to sit down and set specific goals for their child by looking at visuals, and they learned to create the e-book the way they want it you know they take photos and visuals of their child’s activities and view them. And when they see their child making progress, you know it makes them feel proud and that provides a sense of achievement for them, for the family.”*

#### Theme 2: We’ve Got This

The second theme that arose was *we’ve got this*. This appeared across two sub-themes: (1) being in a different place and (2) the importance of self-care.

##### “I Was in This Place, but Now I’m in a Different Place”

The first sub-theme reflected the intervention being a turning point for participants, aptly reflected by one parent saying: “*I was in this place, but now I’m in a different place*.” Both the e-books and to a lesser extent the parent/carer focus group suggested that the intervention helped participants to understand that they make the biggest difference to their child’s positive outcomes, rather than assuming that the professionals know best or that progress is attributed to the expertise of the professional. The process of documenting their progress throughout the interventions demonstrated that they could make change themselves guided by their vision and goals. For example, one e-book noted:

*“I changed as a mum. I have more power over my actions. I am the boss. I have to be myself. I have more quality of life. I enjoy meals with my family together. I have some quality time with myself. Because I changed, the children changed as well. I’m able to set boundaries with my children. I can see different angles with everything. I can see the big picture now. This is about my family. I realize it is not about just my child, it is about all my children and my family.”*

Parents/carers acknowledged, understood, and valued their own knowledge about their children and family more broadly. For instance, one parent noted:

*“For me it’s helped me and my kids to change how we live … for me to see these pictures in front of you to make you think …. it taught me to set something for your life and for your children … I realized the whole picture, what I need to do.”*

The e-books and parent/carer focus group also pointed to a sense of empowerment that occurred through the intervention. Every e-book used the word *power*, *powerful,* or expressed empowerment as an outcome that parents experienced. For instance, one e-book noted: “*I believe in myself. I can see my skills as a powerful mother … I have power because I found power inside me and that will stay with me forever*.” Family focus groups also described the changes they saw in themselves as part of the program.

The professionals also saw changes occurring in the parents. One professional noted:

*“We have very positive outcomes because they (the families) see a constructive and a positive feature. Instead of saying I can’t do that with my child they say, OK I can do this. And there are so many wins-wins and it’s capacity building because the families lead the process, we are just the facilitator.”*

However, the professional focus group data did not highlight the transformative moments, nor the sense of power and empowerment demonstrated by the parents/carers. The e-book might have captured a shift in parents’/carers’ mindsets that occurred through the process of developing their own e-book, which might not have occurred for the professionals facilitating use of the e-books.

##### Looking After Myself Is Important

A second sub-theme focused on recognizing the value of and importance of self-care. Although most parents/carers selected goals that addressed their own wellbeing, they also identified that there were challenges with finding time to focus on these, with a tendency toward prioritizing the need of their child and family over their own needs. Through the intervention, parents/carers recognized that their wellbeing could influence their children. For example, one e-book noted: “*When I look after myself I have more support to focus on the boys. I can be a good support and role model for my children.*” Another noted “*My child is like a mirror. He will reflect my emotions, it must be coming from me.*”

Parents/carers spoke about changing their lifestyle to relax and reach their personal goals and learning to be calmer and happier which could influence their children and promote the changes they want to see. For instance, one parent reflected upon the decision to change her career:

“*It taught me to set my goal with my children, to see the big picture, to look after myself as well, it was really great … so I’m doing a child care course … And it was really great, great for me to get out of the house, not with all the children, it’s my time to study, my time with different people, talking to other people. It was really great.*”

## Discussion

In this paper, we have suggested that despite advances in disability research and practice, care for young children with disability and developmental delays primarily remains grounded in a deficit-based perspective, driven in part by underlying paradigms that permeate language, diagnoses, funding, and interventions. To illustrate, we identified two existing paradigms within disability care: *disability as a disadvantage* and *experts know best*. Then, using a case study approach, we examined the potential for shifting participants’ perspectives of disability care through a SIPP-informed program.

**Figure 1:**
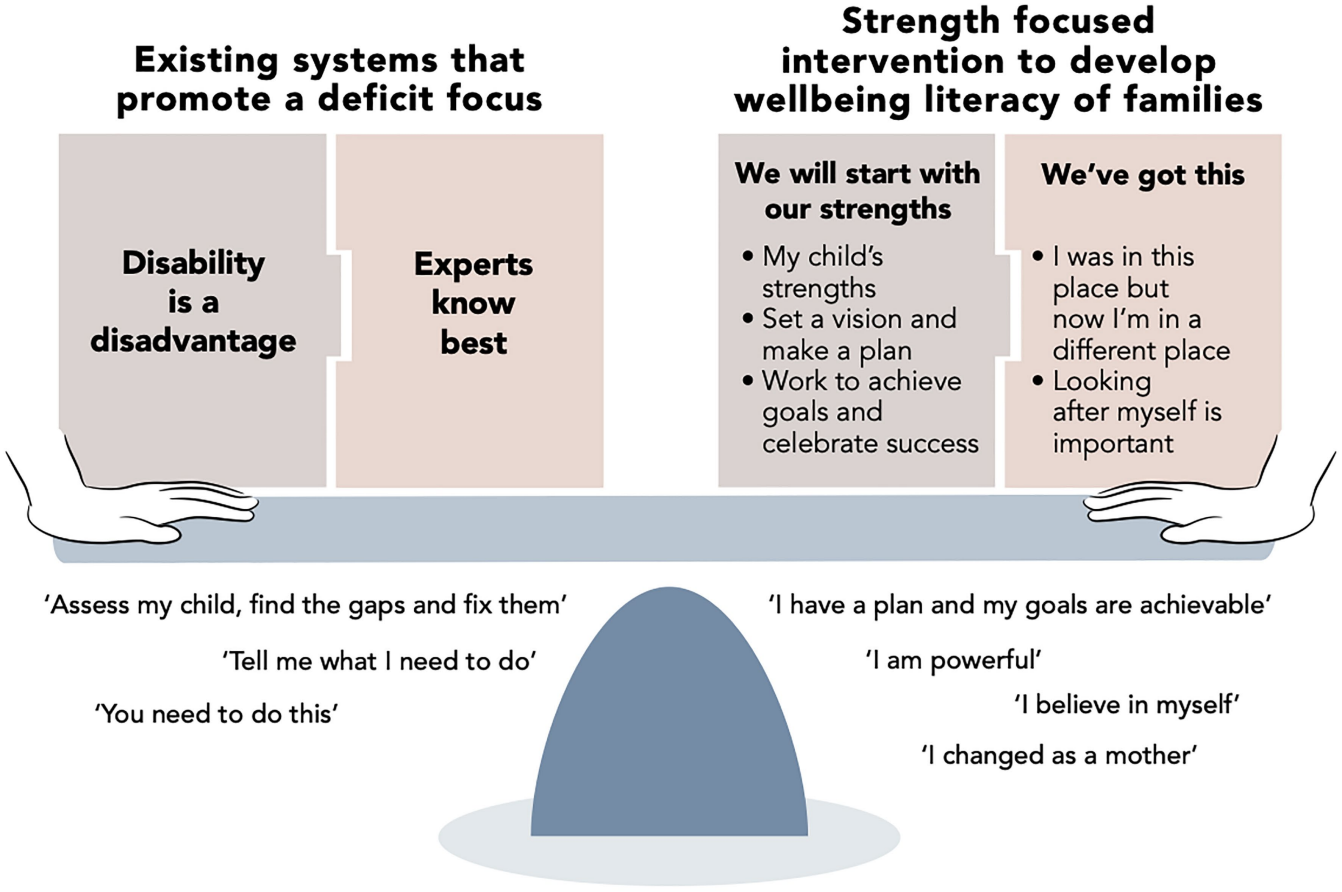
A shift in perspective experienced by participants through the intervention, moving from paradigms of existing systems that promote a deficit focus to strength-based empowerment.

As illustrated in Figure 1, thematic analysis of focus group and e-book data suggested that participants experienced several benefits through the intervention, including a shift in the dominant perspectives through which they experience and approach their child’s disability. Participants developed two primary perspectives: *we will start with our strengths* and *we’ve got this*, reflecting a sense of hope, empowerment, potential, inclusion in the community, and greater independence from the broader system. They demonstrated a growing ability to assist, define, and co-design the services that they need. How might these changes occur? We turn to considering potential mechanisms, implications, and future directions for research and practice.

### Potential Mechanisms of Change

Several mechanisms might be driving shifts in perspective for families and professionals. We suggest that language is critical both for revealing existing paradigms and for shifting those paradigms, which in the current program specifically occurred through the incorporation of the e-books. Here, we focus on two possible mechanisms: the design of the intervention itself and the development of wellbeing literacy.

#### A SIPP-Informed Program

A first potential mechanism is the design of the intervention, which was built upon SIPP principles and co-created with families, professionals, and researchers. The intervention helped parents set and achieve goals, with comments reflecting greater self-efficacy, all important aspects of developing hope and vision for the future (Snyder, 1994). An important part of the process was the inclusion of the novel planning tool and the e-books, which helped families develop greater self-efficacy. This could be because they generated the goals themselves, because they were in their own words and their own language, or because they were recorded in the e-books and regularly reviewed at each session. Indeed, families were far more positive about the e-books than we anticipated. Parents/carers desired action but were not sure where to start, as they had so many goals that they wanted to address to support the positive outcomes for their child’s development. The e-books gave them a process that helped them to resolve these issues.

The tools included in the intervention helped families to relabel and make sense of emotions – developing better emotional literacy. For instance, words such as sorrow and overwhelm describe emotions, but cognitions around those emotions depend on the person’s perceptions of the situation (Ellsworth and Scherer, 2003). The e-books provided a place where families could express themselves using a variety of linguistic and non-linguistic ways. The role of photographs, recordings, and videos in addition to text created evidence for families of their progress at a time when they were influenced by the deficit approach to their child’s diagnosis. Wellbeing arises from positive practices, and the e-books supported the development of those practices.

The e-books particularly appeared to create a sense of empowerment. When families experience the “I’ve got this” moment, a fundamental shift occurs, and they become as Meadows (1999) describes “radically empowered.” Rather than seeing themselves as disempowered observers of their child’s care, parents/carers could see and articulate that with the right tools, they could see themselves as well-functioning people in charge of their own child’s care. The e-books allowed parents to better understand that they play a central role in creating a positive future for their child, both now and into the future. It involves not only focusing on their child’s development, but also on their own wellbeing, enabling sustainable care for their child. As such, the e-books potentially are a useful tool for supporting shared-decision-making, which is considered a fundamental capacity-building strategy in family-centered early childhood intervention (Keen, 2007; Dunst et al., 2019).

Interestingly, empowerment did not appear in the professionals’ focus group data. One explanation for this could be that the child has been traditionally seen as the focus of intervention and that the feelings and experiences of the family are seen as a secondary priority. This reinforces the need for family-centered interventions. Alternatively, while the sense of empowerment was apparent in the 10 e-books selected for analysis, it might not have occurred across the 51 families who participated in the full research project. Still, the sense of empowerment also consistently appeared in the interviews with 14 parents/carers, offering some evidence that empowerment was a consistent theme for parents/carers.

#### The Development of Wellbeing Literacy

A second potential mechanism is the development of wellbeing literacy that occurred through the program. Wellbeing literacy refers to “the vocabulary, knowledge and skills that may be intentionally used to maintain or improve the wellbeing of oneself or others” (Oades et al., 2017, p. 1). Wellbeing can be defined objectively, in terms of the objective conditions of people’s lives, or subjectively, in terms of how people think about, experience, and emotionally evaluate their circumstances (Forgeard et al., 2011; Chia et al., 2020). Here, we focus on subjective aspects, defining wellbeing as feeling and functioning well across multiple domains (Huppert and So, 2013). Thus, wellbeing literacy reflects one’s understanding of, knowledge of, and skills related to cultivating positive functioning in the self and/or others.

The *disability as a disadvantage* paradigm reflects low levels of wellbeing literacy, in that there is a lack of understanding and language around wellbeing, corresponding with a lack of approaches to cultivate wellbeing. Language focuses on deficit and dysfunction, resulting in thoughts and actions focused on decreasing dysfunction. Strength-based approaches to disability reflect high levels of wellbeing literacy, with labels and language reflecting positive aspects of the child and family, resulting in thoughts and actions focused on increasing optimal functioning.

We suggest that the development of wellbeing literacy through interventions such as the e-books used in the current study provides a lens through which parents and peer-groups can voice their needs and preferences, build their decision-making capabilities, and exercise their choice and control. Considering that “wellbeing literacy is how we control the use of wellbeing language” (Oades and Johnston, 2017, p. 2), we suggest that low wellbeing literacy drives the paradigms of disability care that do not serve most young people with disability nor their families well. The language used by individuals, communities, and practitioners points to the underlying paradigms around wellbeing. Shifting paradigms begins with identifying whether language does indeed reveal low levels of wellbeing literacy. Then, the field of positive psychology has developed numerous approaches to cultivate wellbeing-related skills, providing opportunities for the development of wellbeing literacy, with the potential for enabling more optimal outcomes for families, as was demonstrated in our case study.

Research and theory around wellbeing literacy have only recently arisen (*cf.* Oades and Johnston, 2017). While the extent to which participants indeed developed greater wellbeing literacy, that this is a mechanism driving beneficial changes, and that the development of wellbeing literacy can shift individual or collective perspectives and paradigms is unknown; however, our case study provides intriguing possibilities for future research to explore.

### Implications and Future Directions

Our case study results suggest that the e-books provided a process for surfacing and shifting underlying paradigms for the parents. This becomes an important entry point for leveraging change within disability care. Still, while change within individual families is a necessary starting point, this change needs to spread throughout the disability care system for it to take hold. The existing paradigms of *disability as a disadvantage* and *experts know best* are continually reinforced by how experts are trained, how funding schemes are designed, and what permeates throughout approaches to care (Glasby et al., 2009; Duffy, 2010; Kendrick, 2011). As governments around the world have realized, the prevailing mindset of *experts know best* is costly, suffers from workforce shortage, and is unsustainable (Productivity Commission, 2012; Miller and Hayward, 2017; Mavromaras et al., 2018). Most importantly, it disempowers people with disability and their families. Continuing to define *disability as a disadvantage* pathologizes the individual with disability and the family. In contrast, beginning from a place of strength opens unimagined potential of what the person and future hold (Kern et al., 2020). Broader changes are needed, which address the problematic paradigms permeating disability care, with flow on implications for structures, policies, and feedback mechanisms.

Parents, families, and other caregivers play a critical role in the care and support of children and young people with disability and developmental delays. However, in Australia and many places worldwide, current systems do not fully capture their contributions and are unable to unleash the power of strong parent-professional teams working together to create good outcomes for children. As families become more empowered, this positions families as agents of collective change, helping to shift existing systems toward more genuinely family-centered approaches that will, in turn, allow children to exercise choice and control as they develop into adults.

The capacity-building intervention described here appeared to create a turning point for the participants, shifting their mindsets around disability and their role in their child’s care. Participants could recognize paradigms of care that emphasize *disability as a disadvantage* and *experts know best*, benefitting from the alternative and more empowering perspectives of *we will start with our strengths* and *we’ve got this*. Revealing existing paradigms are a first step toward shifting those paradigms, as the invisible becomes visible (Meadows, 1999). But it is unknown the extent to which participating in an intervention and experiencing mental shifts is sufficient, especially when many of the processes, policies, and structures of the broader system remain in the former paradigm.

What does this suggest for system design? At the very least, system structures and policies should not undermine the alternative perspectives that families develop through the development of wellbeing literacy. But more broadly, there is a need to further reveal unhelpful paradigms and the cascading impact that these paradigms have on training, care, policies, funding, and practices. This could happen, for instance, through a social movement with a large groundswell. Families could learn wellbeing literacy skills through modalities such as the planning tool and e-books used in the current intervention, supported by SIPP oriented programs, and then could contribute to the growth of other families through the trust and connection provided by peers.

Once trained in wellbeing literacy, peers could become leaders and teach other families practical approaches for taking action, aligned with the efficacy, wellbeing, and quality of life benefits that reviews suggest arise from interventions that contain a significant peer support component (Shilling et al., 2013; Lancaster et al., 2021). Together, these peer workers might develop a collective benefit mindset that views disability from a positive perspective and encourages social contribution (Buchanan and Kern, 2017; Janson et al., 2018). As more families experience personal mind shifts, they join ranks with a growing number of families who learn from one another that they can make a positive change for the future of both their child and family, ultimately creating collective mind shifts. When these numbers hit a critical mass, change can become widespread, creating a tipping point and transforming disability care.

There is a need to consider the family’s entire journey across the child’s development, beginning in the early years and first diagnosis, and carried across childhood and into adulthood. These experiences include assessments and meetings where professionals gather information about gaps in development, aim to build rapport, or gather information about daily routines with the purpose of identifying priorities for intervention based on family identified needs. Intentional attention needs to be given to the language used throughout all parts of care, identifying words that are empowering versus disempowering, hope-inspiring versus hope-defeating, and strength-based versus deficit-focused. SIPP programs that are strength-based and empower parents, such as the intervention described in this case study, need to become central to disability care. Further, research is needed in terms of how to bridge research and practice.

There is also a need for greater consideration of directly incorporating child voice and participation in both the development and delivery of programs and care. Our case study arose from data collected as part of action research, resulting in an intervention co-designed by parents/carers, professionals, and researchers. The intervention aimed to identify and value the child’s strengths, but the children themselves were not included in the design itself. The inclusion of the child’s voice is increasingly being considered as possible and necessary from the earliest possible age (Carroll and Twomey, 2020; Parsons et al., 2020; Rix et al., 2020). For instance, our experiences here have led to the development of the *Child Voice* model and program (Mahmic and Janson, 2019). Future work should continue to consider strategies for including the entire family within the care process – including parents/carers, siblings, and the children with disability themselves.

### Limitations

While families in our study experienced a new way of capability building through wellbeing literacy, care should be taken in generalizing the results. Replication, incorporating quantitative methods, and expanding to diverse samples are necessary to consider the extent to which the themes identified here generalize. Further testing of the intervention approach, especially the use of the e-books as an approach toward developing wellbeing literacy, is needed, extended to much broader samples, testing both perceived and objective short- and long-term outcomes for families.

As noted, we weighted the e-book data more heavily than the focus group data, since they might be representative of the broader group, beyond the smaller set that were willing to be interviewed. However, even though these were randomly selected, all linguistic elements were in English, which might not be representative of participants who included other languages as part of their e-book entries. We also only analyzed 10 of the 51 available e-books. Full analysis would add several additional considerations, including translation issues (e.g., translating first and then identifying themes or identifying themes in the native language and then finding commonalities) and the amount of data to analyze, which are better suited to supervised machine learning approaches, such as natural language processes (Kern et al., 2016; Eichstaedt et al., 2021).

Although families and practitioners were part of the co-design process, children were not included. Future efforts should consider strategies for effectively incorporating the child voice and participation in the design and delivery of strength-based programs. Aligned with the “call for qual” in positive psychology (Hefferon et al., 2017, p. 211), our study provides an in-depth consideration of a small number of individuals, with the benefit of providing deeper understanding of paradigms within disability care.

## Conclusion

Parents/carers face numerous challenges through diagnosis and decisions around care for children with disability. For better or worse, underlying paradigms around disability impact upon research, practice, policies, funding, and outcomes for not only the child, but for the family and community as a whole. Through a capacity-building intervention that involved interactive tools based upon SIPP principles, our study suggests that change can occur for individuals, as they develop greater wellbeing literacy and gain a sense of empowerment. This can help to “shift our thinking from achieving short term gain through an external intervention done to someone to exploring language-mediated co-created actions which may create ongoing sustained wellbeing gains” (Oades and Johnston, 2017, p. 2). By reconstructing the paradigms of disability, there is greater potential for supporting the optimal development and function of *all* individuals within our human social systems, regardless of ability or background.

## Data Availability Statement

To protect the privacy of participants, the data presented in this article are not readily available. De-identified data can be made available, with restrictions. Requests to access the data should be directed to Peggy.Kern@unimelb.edu.au.

## Ethics Statement

The studies involving human participants were reviewed and approved by Office of Research Services, Western Sydney University (Protocol #H9717). The participants provided their written informed consent to participate in this study.

## Author Contributions

SM: conceptualization, methodology, formal analysis, and project administration. SM, MK, and AJ: writing – original draft preparation and writing – review and editing. All authors contributed to the article and approved the submitted version.

## Funding

This research was supported in part by the Melbourne Disability Institute (2020 Seed Funding Round 2) and Plumtree Learning.

## Conflict of Interest

The authors declare that the research was conducted in the absence of any commercial conflicts of interest. The planning tool used within this study became commercially available after the completion of the research.

## Publisher’s Note

All claims expressed in this article are solely those of the authors and do not necessarily represent those of their affiliated organizations, or those of the publisher, the editors and the reviewers. Any product that may be evaluated in this article, or claim that may be made by its manufacturer, is not guaranteed or endorsed by the publisher.

## Footnotes

^1^see https://sites.google.com/view/frontiers-article/pictability

^2^To protect participant identity and align with procedures approved by the Ethics Committee, e-books cannot be made publicly available. Select parts may be accessible upon request from the corresponding author.

^3^To protect participant identity and align with procedures approved by the Ethics Committee, interview transcriptions cannot be made publicly available. Select parts may be accessible upon request from the corresponding author.
