# Supplementary material for: Identifying and Shifting Disempowering Paradigms for Families of Children With Disability Through a System Informed Positive Psychology Approach
Source: Front Psychol. 2021 Dec 23;12:663640. doi: 10.3389/fpsyg.2021.663640 (PMC8734639; doi:10.3389/fpsyg.2021.663640)

Supplementary Material

1 Focus Group Questions

1.1 Family Focus Groups

1. The Now and Next program starts with planning to help you prioritise what is important for your child and family. Planning also helps everyone work together to achieve your goals. Here is the Pictability planning tool (show Pictability).
    a. Could you talk about your experience of planning using Pictability in Now and Next?
    b. What happened?
    c. What was most useful?
    d. What was least useful?
    e. How does planning with Pictability compare with your other experiences of planning for your child and family?
    f. Is there something you would change about the way we plan together?
2. Families have told us that they need information to make the best decisions for their child. In the e-Book, we created a chapter called Gathering Information. Did this help you gather:
    a. Information for your child?
    b. Information for yourself and your family?
    c. Any other information that was helpful to you?
3. In Now and Next, the e-Book is used to help structure and record your progress.
    a. How did you find using the e-Book?
    b. Did it help you keep track of your goals?
    c. What were the good things about using the e-Book?
    d. Was there anything you didn't like about using the e-Book?
4. Families have told us that they need information to help them make the best decisions in spending their individual funding.
    a. Did any information help you to make decisions about your Individual Funding? If yes, what and how?
    b. Did the e-Book help you make the best decisions in spending the individual funding? If yes, how?
5. In each session you were asked to reflect on your learning and then you record this in your e-Book. Reflecting on your learning was then used to create 'Mottos' that summarised what you learnt. We would like to know what you think of this experience.
    a. How did you find this process?
    b. Was it helpful? If so, how?
    c. Do you have any suggestions of how we could improve this process?
6. When thinking back to how you felt at the beginning of Now and Next and now, has anything changed for you, your child, your family?
7. What did you learn from the Now and Next process?
8. Families also receive an individual funding package to help them meet their goals.
    a. How have you used your individual funding?
    b. Did it make a difference to your child? How?
    c. Did it make a difference to your family? How?

9. What was the best thing about Now and Next?
10. If there was one thing that you could change about Now and Next, what would it be?
11. Are there any comments you would like to make?

1.2 Professional Focus Groups

1. The Now and Next program starts with planning to help families prioritise what is important to them and their child. Planning also helps everyone to work together to achieve the goals. Here is the Pictability planning tool (show Pictability).
    a. Could you talk about your experience of planning with Pictability in Now and Next?
    b. What happened?
    c. What was most useful?
    d. What was least useful?
    e. How does planning with Pictability compare with your other experiences of planning?
    f. Is there something you would change about the way you plan together with families?
2. Families have told us that they need information to make the best decisions for their child. In the e-Book, there is a chapter called Gathering Information. Here is an example of an e-Book. (show e-Book). Did this help you gather information:
    a. For the child? Yes/No. Please explain.
    b. For the family? Yes/No. Please explain.
    c. Are there any other ways we can help families to get information that is tailored specifically for them?
3. In Now and Next, we used the e-Book to helps structure and record progress.
    a. How did you find using the e-Book?
    b. Did it help you and the family keep track of the goals?
    c. What were the good things about using the e-Book?
    d. Was there anything you didn’t like about using the e-Book?
4. Families have told us that they need information to help them make the best decisions in spending their individual funding.
    a. What information helped you to understand about individual funding?
    b. What information about individual funding was helpful to families?
    c. Did the e-Book help? If so, how?
5. In each session families are asked to reflect on their learning and then record this in their e-Book. This process is then used to facilitate guided reflective practice for families and collect ‘Mottos’ that summarise what families learnt.
    a. How did you find this process?
    b. Did your experience of helping families reflect and record their progress change over time?
    c. Do you have any suggestions for how we could improve this process?
6. When thinking back to how you felt at the beginning of Now and Next and now, has anything changed?
7. Families also receive an individual funding package to help them meet their goals.
    a. Has this made a difference to children?
    b. Has this made a difference to families?
    c. Has individual funding influenced the way your work with families? If so, how?
8. Now and Next is a new program and your contribution was important to this process. Has Now and Next changed:

a. How you relate to families?
b. The way you work with families?
c. How you spend your time during the session and back at the office?
9. What was the best thing about Now and Next?
10. If there was one thing that you could change about Now and Next, what would it be?
11. Are there any comments you would like to make?

.

2 Sample screenshots from the e-books

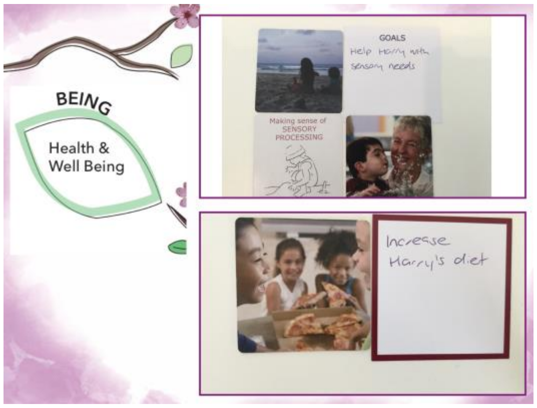

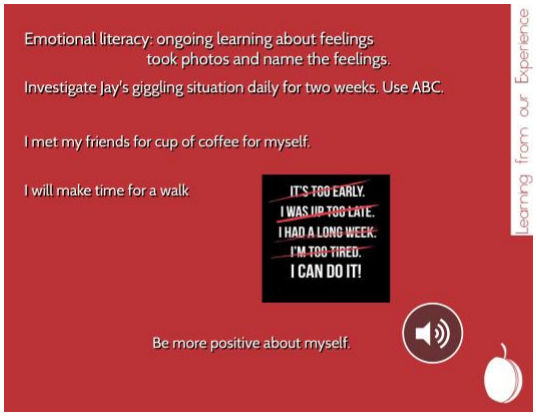

Supplement: Supplementary file 1 [file Data_Sheet_1.pdf]
